# Supplementary figures and images for: Cross-reactivity of rPvs48/45, a recombinant Plasmodium vivax protein, with sera from Plasmodium falciparum endemic areas of Africa
Source: bioRxiv. 2024 Apr 15:2024.04.10.588966. Preprint. [Version 2] doi: 10.1101/2024.04.10.588966 (PMC11042229; doi:10.1101/2024.04.10.588966)

## Slide 1
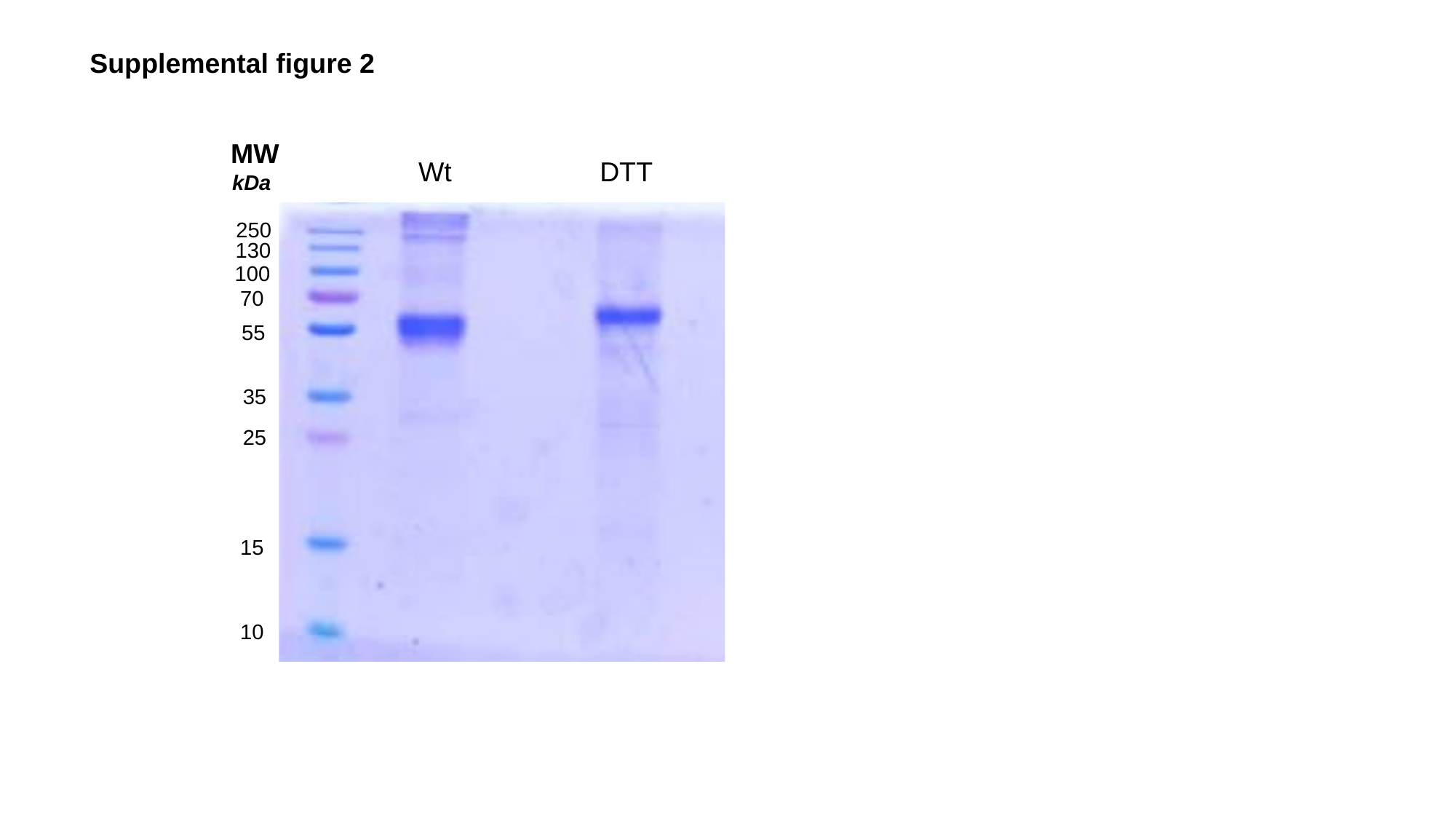

Supplemental figure 2
MW
kDa
250
130
100
70
55
35
25
15
10
Wt
DTT

Supplement: Supplement 1 — Supplemental figure 2: Recombinant CHO-rPvs48/45 protein analysis in western Blott CHO-rPvs48/45 protein identity was confirmed using 12% SDS-PAGE gel in western bloot. Analysis was carried out under reducing (0.05 mol/L dithiothreitol, DTT) and non-reducing conditions (wt) [55]. [file media-1.ppt]
