# Supplementary material for: Cross-reactivity of rPvs48/45, a recombinant Plasmodium vivax protein, with sera from Plasmodium falciparum endemic areas of Africa
Source: bioRxiv. 2024 Apr 15:2024.04.10.588966. Preprint. [Version 2] doi: 10.1101/2024.04.10.588966 (PMC11042229; doi:10.1101/2024.04.10.588966)
Supplement: Supplement 2 — Supplemental figure 1. Sequence homology between the Pvs48/45 and Pfs48/45 proteins The amino acid (aa) sequences alignment of full-length Pvs48/45 and Pfs48/45 proteins were obtained using PlasmoDB database, and sequences matched with Blastp (protein-protein BLAST; https://bit.ly/3C0hPpK). Pfs48/45 and Pvs48/45 share ~56% identity (238 out of 423) and ~78% similarity in their protein sequences. Conserved cysteine residues are identified by C letter in black and bold. In the red sequence, identical amino acid residues are identified by (letter|), the similar residues by (+) and the different residue by (*). [file media-2.pptx]

## Slide 1
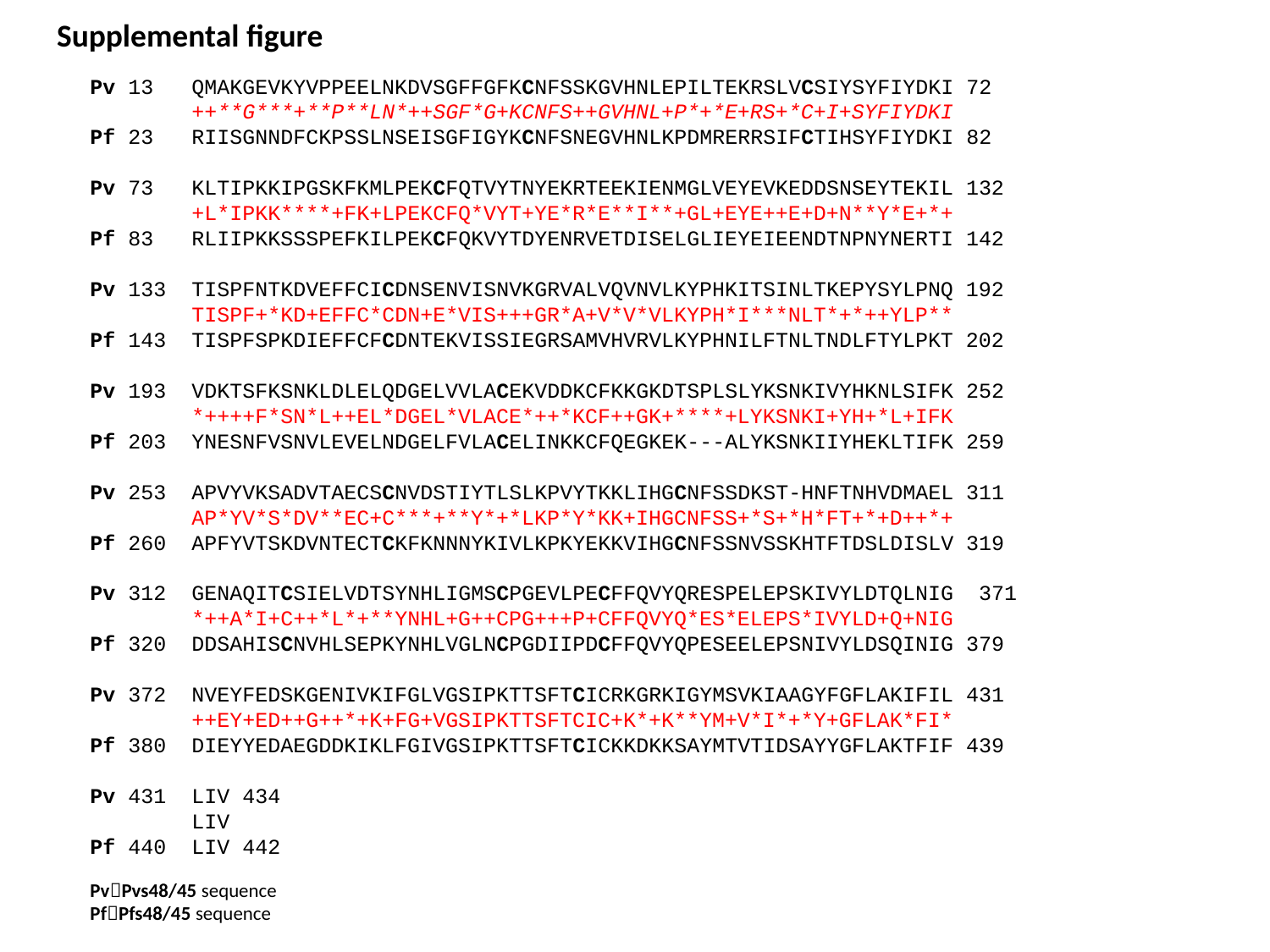

Supplemental figure
Pv 13 QMAKGEVKYVPPEELNKDVSGFFGFKCNFSSKGVHNLEPILTEKRSLVCSIYSYFIYDKI 72
 ++**G***+**P**LN*++SGF*G+KCNFS++GVHNL+P*+*E+RS+*C+I+SYFIYDKI
Pf 23 RIISGNNDFCKPSSLNSEISGFIGYKCNFSNEGVHNLKPDMRERRSIFCTIHSYFIYDKI 82
Pv 73 KLTIPKKIPGSKFKMLPEKCFQTVYTNYEKRTEEKIENMGLVEYEVKEDDSNSEYTEKIL 132
 +L*IPKK****+FK+LPEKCFQ*VYT+YE*R*E**I**+GL+EYE++E+D+N**Y*E+*+
Pf 83 RLIIPKKSSSPEFKILPEKCFQKVYTDYENRVETDISELGLIEYEIEENDTNPNYNERTI 142
Pv 133 TISPFNTKDVEFFCICDNSENVISNVKGRVALVQVNVLKYPHKITSINLTKEPYSYLPNQ 192
 TISPF+*KD+EFFC*CDN+E*VIS+++GR*A+V*V*VLKYPH*I***NLT*+*++YLP**
Pf 143 TISPFSPKDIEFFCFCDNTEKVISSIEGRSAMVHVRVLKYPHNILFTNLTNDLFTYLPKT 202
Pv 193 VDKTSFKSNKLDLELQDGELVVLACEKVDDKCFKKGKDTSPLSLYKSNKIVYHKNLSIFK 252
 *++++F*SN*L++EL*DGEL*VLACE*++*KCF++GK+****+LYKSNKI+YH+*L+IFK
Pf 203 YNESNFVSNVLEVELNDGELFVLACELINKKCFQEGKEK---ALYKSNKIIYHEKLTIFK 259
Pv 253 APVYVKSADVTAECSCNVDSTIYTLSLKPVYTKKLIHGCNFSSDKST-HNFTNHVDMAEL 311
 AP*YV*S*DV**EC+C***+**Y*+*LKP*Y*KK+IHGCNFSS+*S+*H*FT+*+D++*+
Pf 260 APFYVTSKDVNTECTCKFKNNNYKIVLKPKYEKKVIHGCNFSSNVSSKHTFTDSLDISLV 319
Pv 312 GENAQITCSIELVDTSYNHLIGMSCPGEVLPECFFQVYQRESPELEPSKIVYLDTQLNIG 371
 *++A*I+C++*L*+**YNHL+G++CPG+++P+CFFQVYQ*ES*ELEPS*IVYLD+Q+NIG
Pf 320 DDSAHISCNVHLSEPKYNHLVGLNCPGDIIPDCFFQVYQPESEELEPSNIVYLDSQINIG 379
Pv 372 NVEYFEDSKGENIVKIFGLVGSIPKTTSFTCICRKGRKIGYMSVKIAAGYFGFLAKIFIL 431
 ++EY+ED++G++*+K+FG+VGSIPKTTSFTCIC+K*+K**YM+V*I*+*Y+GFLAK*FI*
Pf 380 DIEYYEDAEGDDKIKLFGIVGSIPKTTSFTCICKKDKKSAYMTVTIDSAYYGFLAKTFIF 439
Pv 431 LIV 434
 LIV
Pf 440 LIV 442
PvPvs48/45 sequence
PfPfs48/45 sequence
